# 

**Published:** 1906-02-10

**Authors:** 


### NOTICE TO CORRESPONDENTS.

All MSS., Letters, Books for Review, and other matters intended for the Editor should be addressed THE EDITOR, "THE HOSPITAL," THE HOSPITAL BUILDINGS, 28 & 29 SOUTHAMPTON STREET, STRAND, W.C.

The Editor cannot undertake to return rejected MS., even when accompanied by stamped directed envelope.

**Notice to Advertisers and Subscribers.**

All Advertisements, Orders for Copies of THE HOSPITAL, and Business Communications should be addressed to **THE MANAGER (Not to the Editor),** THE HOSPITAL, 28 & 29 Southampton Street, Strand London, W.C.

The Cost of Subscription, post free, is as follows:—Per week, 3½d.; per quarter, 3s. 3d.; per half-year, 6s. 6d.; per annum, 13s. Foreign and Colonial:—Per annum, 19s. 6d., payable, in all cases, in advance.

**NOTICE.—Vol. XXXVIII. of "The Hospital" April to September 1905), in handsome cloth binding, will shortly be on sale at the office of this Journal, price 10s. 6d. Cases for binding the Numbers contained in this Volume 2s. Index to Vol. XXXVIII., 3½d., post free.**

**The Monthly Part for February is now ready. Price 1s., by post, 1s. 3d.**

### BOOKS RECEIVED.

A. and C. Black.
"The Writers' and Artists' Year-book, 1906."
"Englishwoman's Year-book, 1906."

Local Government Journal Office.
"Departmental Decisions."
"The Water Supply of Villages and Small Towns."
"Local Government Annual, 1906."

The Scientific Press, Ltd.
"Instructions to Midwives."

Smith, Elder and Co.
"Bodily Deformities." By E. J. Chance, F.R.C.S. Edited by J. Poland, F.R.C.S.

The Author.
"A Lecture upon Massage." By H. Tibbits, M.D.

### MEDICAL APPOINTMENTS AND VACANCIES.

### VACANCIES.

Cancer Hospital, Fulham Road, London, S.W.—Senior House Surgeon.

Canterbury, Kent, and Canterbury Hospital.—House Physician, also House Surgeon.

Central Midwives Board.—Examiners.

City of London Hospital for Diseases of the Chest, Victoria Park, E.—Two Physicians to Out-patients.

Durham County Council.—Inspector of Midwives and Health Visitor (female).

Guildford, Royal Surrey County Hospital.—Assistant House Surgeon.

Hospital for Sick Children, Great Ormond Street, London, W.C.—Assistant Surgeon.

Larbert, Stirling District Asylum.—Assistant Medical Officer.

Leicester Infirmary.—Assistant House Physician.

London County Council.—Medical Officer to Employés in the Boroughs of Hammersmith and Fulham.

Lincoln County Hospital.—Senior House Surgeon.

Manchester, Ancoats Hospital.—Resident House Surgeon. Also Resident House Physician.

Manchester Hospital for Consumption and Diseases of the Throat and Chest.—Honorary Surgeon.

Metropolitan Hospital, Kingsland Road, N.E.—Assistant Physician.

Milton and Sittingbourne.—Medical Officer of Health.

Newcastle-on-Tyne Dispensary.—Two Visiting Medical Assistants.

Oxford, Radcliffe Infirmary.—House Physician and Junior House Surgeon.

Royal Free Hospital, Gray's Inn Road, W.C.—Anæsthetist and Curator of Museum (female).

Wolverhampton and Staffordshire General Hospital.—Assistant House Surgeon.

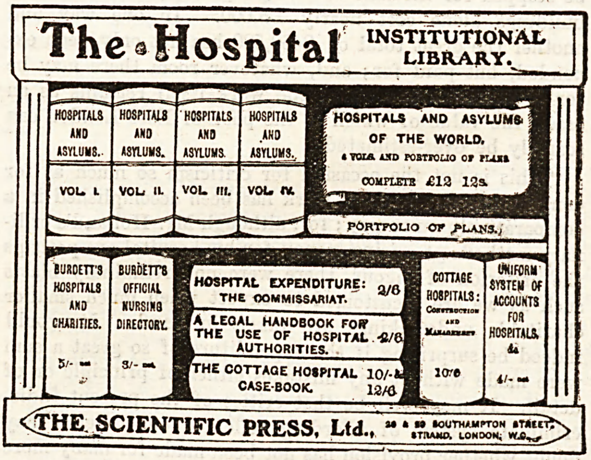

### Medical Appointments.

THE

**CANCER HOSPITAL (FREE),**

**FULHAM ROAD, LONDON, S.W.**

Applications are invited for the post of SENIOR HOUSE-SURGEON, who must be qualified under the Medical Registration Act. Appointment for six months. Salary £80 per annum with Board and Residence. Applications, with copies of testimonials, to be forwarded to the Secretary on or before February 12.

FRED. W. HOWELL, Secretary.

(936)

### WRITE! FOR A COMPLETE CATALOGUE OF NURSING MANUALS.

IT WILL INTEREST YOU.

### A Few Books on Special Subjects.

### Diet

**ART OF FEEDING THE INVALID.**
New and Popular Edition. With Special Chapter on Feeding in Consumption. 1/6 post free.

**DIET IN SICKNESS AND IN HEALTH.**
By Mrs. Ernest Hart. With an Introduction by Sir Henry Thompson, F.R.C.S., M.B.Lond. Illustrated, 3/6 post free.

**A PRACTICAL GUIDE TO COOKERY IN WEST AFRICA AND IN THE TROPICS.**
By Sister Cockburn, late Colonial Nursing Service, Sierra Leone, West Africa. 3/- net, 3/3 post free.

### Surgical Work

**SURGICAL INSTRUMENTS AND APPLIANCES USED IN OPERATIONS.**
A Classified List, Illustrated with Explanatory Notes. 1/6 net, 1/8 post free.

**SURGICAL WARD-WORK AND NURSING.**
By Dr. Miles. 3/6 net, 3/9 post free.

**ON PREPARATION FOR OPERATION IN PRIVATE HOUSE.**
By Stanmore Bishop, F.R.C.S. 6d. net, 7d. post free.

### Fevers

**THE NURSING OF INFECTIOUS DISEASES.**
By E. J. Woollacott, M.A., M.D. 2/6 net, 2/9 post free.

**FEVERS AND INFECTIOUS DISEASES.**
The Nursing and Practical Management.
By Wm. Harding, M.D. 1/- post free.

**HINTS TO NURSES ON TROPICAL FEVERS.**
By Sister Pollard. 1/6 net, 1/8 post free.

### Physiology and Anatomy

**ELEMENTARY ANATOMY AND SURGERY FOR NURSES.**

By Wm. McAdam Eccles, M.S. Lond. Illustrated, 2/6 post free.

**ELEMENTARY PHYSIOLOGY FOR NURSES.**
By C. F. Marshall, M.D. 2/- post free.

**ELEMENTS OF ANATOMY AND PHYSIOLOGY.**
By W. Bernard Secretan. Illustrated, 2/- net, 2/3 post free.

**The Scientific Press, Ltd.**

PUBLISHERS & BOOKSELLERS.
Nursing Manuals, Case-Books, and Charts of all Descriptions.
28 & 29 SOUTHAMPTON STREET, STRAND, LONDON, W.C.

